# Association of Tumor Protein p53 and Ataxia-Telangiectasia Mutated Comutation With Response to Immune Checkpoint Inhibitors and Mortality in Patients With Non–Small Cell Lung Cancer

**DOI:** 10.1001/jamanetworkopen.2019.11895

**Published:** 2019-09-20

**Authors:** Yu Chen, Gang Chen, Jin Li, Ying-Ying Huang, Yi Li, Jing Lin, Li-Zhu Chen, Jian-Ping Lu, Yu-Qi Wang, Chang-Xi Wang, Leong Kin Pan, Xue-Feng Xia, Xin Yi, Chuan-Ben Chen, Xiong-Wei Zheng, Zeng-Qing Guo, Jian-Ji Pan

**Affiliations:** 1 Fujian Provincial Key Laboratory of Translational Cancer Medicine, Fuzhou, China; 2 Cancer Bio-immunotherapy Center, Fujian Medical University Cancer Hospital and Fujian Cancer Hospital, Fuzhou, China; 3 Department of Medical Oncology, Fujian Medical University Cancer Hospital and Fujian Cancer Hospital, Fuzhou, China; 4 Department of Pathology, Fujian Medical University Cancer Hospital and Fujian Cancer Hospital, Fuzhou, China; 5 Geneplus-Beijing Institute, Beijing, China; 6 Fujian Medical University Cancer Hospital, Fuzhou, China; 7 China Certification and Inspection Group, Kuok Kim Medical Center III, Macao, China; 8 Hui Xian Medical Center, Macao, China; 9 Department of Radiation Oncology, Fujian Medical University Cancer Hospital and Fujian Cancer Hospital, Fuzhou, China

## Abstract

**Importance:**

Immune checkpoint inhibitors (ICIs) can elicit durable antitumor responses in patients with non–small cell lung cancer (NSCLC), but only 20% to 25% of patients respond to treatment. As important genes in the DNA damage response pathway, comutation in the tumor protein p53 (*TP53*) and ataxia-telangiectasia mutated (*ATM*) genes may be associated with genomic instability and hypermutation. However, the prevalence of *TP53* and *ATM* comutation and its association with response to ICIs are not fully understood.

**Objective:**

To examine the prevalence of the *TP53* and *ATM* comutation, the potential mechanism, and its association with response to ICIs among patients with NSCLC.

**Design, Setting, and Participants:**

This multiple-cohort study included patients with NSCLC from the Geneplus Institute, the Cancer Genome Atlas (TCGA), and the Memorial Sloan Kettering Cancer Center (MSKCC) databases and from the POPLAR and OAK randomized controlled trials. Samples in the Geneplus cohort were collected and analyzed from April 30, 2015, through February 28, 2019. Data from TCGA, the MSKCC, and the POPLAR and OAK cohorts were obtained on January 1, 2019, and analyzed from January 1 to April 10, 2019. Next-generation sequencing assays were performed on tumor samples by the Geneplus Institute. Genomic, transcriptomic, and clinical data were obtained from TCGA and MSKCC databases.

**Exposures:**

Comprehensive genetic profiling was performed to determine the prevalence of *TP53* and *ATM* comutation and its association with prognosis and response to ICIs.

**Main Outcomes and Measures:**

The main outcomes were *TP53* and *ATM* comutation frequency, overall survival (OS), progression-free survival, gene set enrichment analysis, and immune profile in NSCLC.

**Results:**

Patients with NSCLC analyzed in this study included 2020 patients in the Geneplus cohort (mean [SD] age, 59.5 [10.5] years; 1168 [57.8%] men), 1031 patients in TCGA cohort (mean [SD] age, 66.2 [9.5] years; 579 [56.2%] men), 1527 patients in the MSKCC cohort (662 [43.4%] men), 350 patients in the MSKCC cohort who were treated with ICIs (mean [SD] age, 61.4 [13.8] years; 170 [48.6%] men), and 853 patients in the POPLAR and OAK cohort (mean [SD] age, 63.0 [9.1] years; 527 [61.8%] men). Sites of *TP53* and *ATM* comutation were found scattered throughout the genes, and no significant difference was observed in the frequency of *TP53* and *ATM* comutation within the histologic subtypes and driver genes. In 5 independent cohorts of patients with NSCLC, *TP53* and *ATM* comutation was associated with a significantly higher tumor mutation burden compared with the sole mutation and with no mutation (TCGA, MSKCC, Geneplus, and POPLAR and OAK cohort). Among patients treated with ICIs in the MSKCC cohort, *TP53* and *ATM* comutation was associated with better OS than a single mutation and no mutation among patients with any cancer (median OS: *TP53* and *ATM* comutation, not reached; *TP53* mutation alone, 14.0 months; *ATM* mutation alone, 40.0 months; no mutation, 22.0 months; *P* = .001; NSCLC median OS: *TP53* and *ATM* comutation, not reached; *TP53* mutation alone, 11.0 months; *ATM* mutation alone, 16.0 months; no mutation, 14.0 months; *P* = .24). Similar results were found in the POPLAR and OAK cohort in which the disease control benefit rate, progression-free survival, and OS were all greater in patients with the *TP53* and *ATM* comutation compared with the other 3 groups (median progression-free survival: *TP53* and *ATM* comutation, 10.4 months; *TP53* mutation, 1.6 months; *ATM* mutation, 3.5 months; no mutation, 2.8 months; *P* = .01; median OS: *TP53* and *ATM* comutation, 22.1 months; *TP53* mutation, 8.3 months; *ATM* mutation, 15.8 months; no mutation, 15.3 months; *P* = .002).

**Conclusions and Relevance:**

This study’s findings suggest that the *TP53* and *ATM* comutation occurs in a subgroup of patients with NSCLC and is associated with an increased tumor mutation burden and response to ICIs. This suggests that *TP53* and *ATM* comutation may have implications as a biomarker for guiding ICI treatment.

## Key Points

**Question:**

What are the prevalence and association of tumor protein p53 (*TP53*) and ataxia-telangiectasia mutated (*ATM*) comutation with response to immune checkpoint inhibitors in patients with non–small cell lung cancer (NSCLC)?

**Findings:**

In this multiple-cohort study, *TP53* and *ATM* comutation sites were scattered throughout the genes analyzed. Comutation in *TP53* and *ATM* was associated with a higher tumor mutation burden and better overall survival compared with sole mutations and no mutation.

**Meaning:**

Patients with *TP53* and *ATM* comutation compose a subgroup of patients with NSCLC associated with an increased tumor mutation burden and better response to immune checkpoint inhibitors; *TP53* and *ATM* may be a clinically relevant biomarker in guiding immunotherapy treatment of NSCLC.

## Teaser

This cohort study examines the association of comutation of tumor protein p53 (*TP53*) and ataxia-telangiectasia mutated (*ATM*) genes with response to immune checkpoint inhibitor (ICI) treatment and overall survival among patients with non–small cell lung cancer (NSCLC).

## Introduction

Recent developments in immune checkpoint inhibitors (ICIs) have improved the survival in a multitude of advanced malignant neoplasms, including non–small cell lung cancer (NSCLC).^1,2,3,4,5,6^ However, most patients receiving ICIs do not derive a benefit. An important aspect of immunotherapy is how to identify and develop predictive biomarkers of ICI response.^7,8^ The commonly used clinically applicable predictive biomarker has been programmed cell death 1 ligand 1 (PD-L1), also known as *cluster of differentiation 274* or *CD274* (OMIM 605402) determined with immunohistochemistry. The Keynote-024 study^1^ found that the presence of tumor PD-L1 expression more than 50% was associated with the efficacy of pembrolizumab in first-line therapy. However, the sensitivity and specificity of PD-L1 expression are modest,^9^ which has prompted the search for additional tools.^10,11^ Mutation of mismatch repair (MMR) genes is known contribute to damage to the DNA damage response (DDR) pathway, which is associated with an increase in the tumor mutation burden (TMB),^12^ including catalytic subunit of DNA polymerase epsilon (*POLE*) (OMIM 174762) gene; DNA polymerase δ 1, catalytic subunit (*POLD1*) (OMIM 174761); breast cancer susceptibility gene 1 (*BRCA1*) (OMIM 113705); and breast cancer susceptibility gene 2 (*BRCA2*) (OMIM 600185), which are associated with the efficacy of ICIs in treating lung cancer, but the frequency of occurrence among patients with lung cancer is low.^13,14,15^

The tumor protein p53 (*TP53*) (OMIM 191170) tumor suppressor gene encodes the p53 transcription factor and is the most commonly mutated gene in human cancers. Under various cellular stress conditions, p53 is activated to inhibit transformation by inducing cell cycle arrest, DNA damage repair, senescence, or apoptosis.^16^ Loss of ataxia-telangiectasia mutated (*ATM*) (OMIM 607585) function is associated with the autosomal recessive disease ataxia-telangiectasia, cerebellar degeneration, hypersensitivity to ionizing radiation, cancer susceptibility, immunodeficiency, and genomic instability.^17^ Human tumors deficient of ATM frequently display chemotherapy resistance and poor survival.^18^ The aim of this study was to describe an integrative analysis of *TP53* and *ATM* comutation in the Cancer Genome Atlas (TCGA) database,^15^ Geneplus database, Memorial Sloan Kettering Cancer Center (MSKCC) database,^19^ and the POPLAR^4^ and OAK^20^ cohorts to highlight the importance of validation of the *TP53* and *ATM* comutation for the delivery of precision immunotherapy.

## Method

### Patients and Samples

From April 30, 2015, through February 28, 2019, 17 814 patients, including 2020 patients with NSCLC, underwent a next-generation sequencing (NGS) assay in the Geneplus-Beijing Institute, Beijing, China. All procedures were conducted in accordance with the Declaration of Helsinki^21^ and with approval from the ethics committee of Fujian Provincial Cancer Hospital. Written informed consent was obtained from all participants. The study was conducted using the Strengthening the Strengthening the Reporting of Observational Studies in Epidemiology (STROBE) reporting guideline.

### Sequencing and Analysis

Comprehensive genomic profiling for the Chinese cohort was performed using customized panels of 59 genes or 1021 genes (eTable 1 in the Supplement). Details of sample processing, DNA extraction, library preparation, target capture, NGS, and analysis are described in eMethods 1, 2, and 3 in the Supplement.

### NSCLC Data

Somatic mutation data for 11 097 solid tumor samples, including 1031 NSCLC samples, in the TCGA database^15^ and 1527 NSCLC samples in the MSKCC database were downloaded from cBioPortal.^19^ Gene expression data in fragments per kilobase of transcripts per million mapped reads (FPKM) for 969 NSCLC samples in the TCGA database^15^ were obtained from the Broad Institute Genomic Data Analysis Center.^22^

A total of 1662 patients, including 350 patients with NSCLC, treated at MSKCC^23^ received at least 1 dose of ICIs with overall survival (OS) defined from the date of first infusion of any ICI. More characteristics of the patients treated with ICIs in the MSKCC database^23^ are presented in eTable 2 in the Supplement.

For the POPLAR^4^ and OAK^20^ cohort, clinical and somatic mutation data were obtained from a previous study. The POPLAR^4^ and OAK^20^ cohort included data from 853 patients with NSCLC. A total of 429 patients received atezolizumab, while 424 patients received docetaxel. More characteristics of patients who received atezolizumab are presented in eTable 3 in the Supplement. Details about data sources are presented in eFigure 1 in the Supplement.

### Assessment of TMB

The TMB was defined as the number of somatic nonsynonymous variations, insertions, and deletions in examined coding regions detected in tumor tissues by whole-exon sequencing in TCGA^15^ and NGS in the MSKCC^19^ and Geneplus databases. In the MSKCC cohort^19^ of patients with NSCLC, 341 samples underwent targeted NGS (ie, integrated mutation profiling of actionable cancer targets) with a customized panel of 341 genes, while 1186 samples were analyzed with a panel of 410 genes. The TMB was compared among patients who received integrated mutation profiling of actionable cancer targets testing with the same designed panel. In the POPLAR^4^ and OAK^20^ cohort, the TMB was evaluated as somatic nonsynonymous variations, insertions, and deletions detected in blood samples using a companion diagnostic assay (FoundationOne).

### Gene Set Enrichment Analysis

The expression value was transformed by log_2_(FPKM + 1) for further analysis. Based on the hallmark gene sets, Gene Set Enrichment Analysis software version 3.0 (Broad Institute) was used to identify significantly altered gene sets (false discovery rate ≤ 0.10) among the groups. For significantly enriched pathways in the comutated group, single-sample gene set enrichment analysis was used to calculate the enrichment score in individual samples. The rank sum test was performed to evaluate the statistical difference. To measure the relative levels of tumor infiltrating lymphocytes subsets, published signature gene sets were assessed by single-sample gene set enrichment analysis.^24^

### Statistical Analysis

Pearson χ^2^ test or Fisher exact test were used to assess categorical variables. Differences between the 2 groups were examined with 2-tailed unpaired *t* test for normally distributed variables or with the Mann-Whitney test for nonnormally distributed variables. Kaplan-Meier survival and multivariate Cox regression analyses were used to analyze associations between mutation type and survival. Statistical analyses were performed using SPSS statistical software version 23.0 (SPSS) and Prism analysis and graphic software version 8.0.1 (GraphPad). A 2-sided *P* value of less than .05 was considered statistically significant. Data were analyzed from January 1, 2019, to April 10, 2019.

## Results

Our study included 17 814 patients in the Geneplus cohort, including 2020 patients with NSCLC (mean [SD] age, 59.5 [10.5] years; 1168 [57.8%] men). Our study also found 4 cohorts in the literature for analyses, including 1031 patients with NSCLC in the TCGA cohort^15^ (mean [SD] age, 66.2 [9.5] years; 579 [56.2%] men, 398 [38.6%] women, and 54 [5.2%] unknown sex), 1527 patients with NSCLC in the MSKCC cohort^19^ (662 [43.4%] men), 1662 patients in the MSKCC cohort who were treated with ICIs^23^ (mean [SD] age, 61.4 [13.8] years; 1034 [62.2%] men), including with 350 patients with NSCLC (170 [48.6%] men), and 853 patients in the POPLAR^4^ and OAK^20^ cohort (mean [SD] age, 63.0 [9.1] years; 527 [61.8%] men).

### Distribution and Clinical Implications of the *TP53* and *ATM* Comutation Profile Landscape

**Figure 1.**
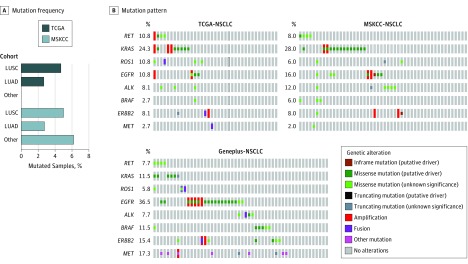
Assessment of Frequency of Tumor Protein p53 and Ataxia-Telangiectasia Mutated Comutation and Mutation Pattern of Driver Genes in Patients With Non–Small Cell Lung Cancer (NSCLC) From 3 Cohorts LUAD indicates lung adenocarcinoma; LUSC, lung squamous cell carcinoma; MSKCC, Memorial Sloan Kettering Cancer Center; and TCGA, The Cancer Genome Atlas.

We found the *TP53* and *ATM* comutation in 37 of 1031 patients with NSCLC (3.6%) in the TCGA database^15^ and 52 of 2020 patients with NSCLC (2.6%) in the Geneplus database (eFigure 2 in the Supplement). Subsequently, we surveyed mutation sites in *TP53* and *ATM* between comutated and singularly mutated samples. The *TP53* and *ATM* mutations were found scattered throughout the genes in comutated samples (eFigure 3 in the Supplement). Moreover, 532 of 1031 patients with NSCLC (54.5%) in the TCGA database^15^ and 1238 of 1527 patients with NSCLC (81.1%) in the MSKCC database^19^ had lung adenocarcinoma. We did not observe significant differences in the *TP53* and *ATM* comutation frequency within the histologic subtypes (Figure 1A). Next, we investigated the mutation pattern of driver genes among patients with NSCLC who had the *TP53* and *ATM* comutation. Similarly, 10.8% of patients in the TCGA cohort,^15^ 16.0% of patients in the MSKCC cohort,^19^ and 36.5% of patients in the Geneplus cohort who had the *TP53* and *ATM* comutation also had epidermal growth factor receptor (*EGFR*) (OMIM 131550) mutations (Figure 1B). Among patients with NSCLC and the *TP53* and *ATM* comutation, 8.1% of patients in the TCGA cohort^15^ and 7.7% of patients in the Geneplus cohort also had anaplastic lymphoma kinase (*ALK*) tyrosine kinase receptor (OMIM 105590) fusion (Figure 1B). Neither concurrent nor exclusive mutation patterns were identified between driver genes and the *TP53* and *ATM* comutation.

### Association of *TP53* and *ATM* Comutation With Increasing TMB

**Figure 2.**
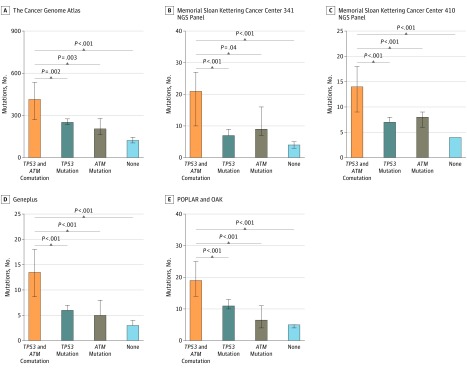
Tumor Mutation Burden of Samples From Patients With Non–Small Cell Lung Cancer From The Cancer Gene Atlas,^15^ Memorial Sloan Kettering Cancer Center (MSKCC),^19^ Geneplus, and POPLAR^4^ and OAK^20^ Cohorts. The height of the bars indicates median value; error bar, 95% CI. *ATM* indicates ataxia-telangiectasia mutated gene; NGS, next-generation sequencing; and *TP53*, tumor protein p53 gene.

To determine whether the *TP53* and *ATM* comutation had a significant association with an increased TMB, we compared the mutation load of samples among patients who had the comutation, the *TP53* mutation alone, the *ATM* mutation alone, or no mutation in 5 independent NSCLC cohorts: TCGA,^15^ MSKCC 341 NGS panel genes, MSKCC 410 NGS panel genes,^19^ Geneplus, and POPLAR^4^ and OAK.^20^ All comparisons indicated that the *TP53* and *ATM* comutation was associated with a significantly higher TMB compared with the other 3 groups in all cohorts. In the TCGA cohort,^15^ the median (interquartile range [IQR]) TMB was 414.0 (207.5-766.0) mutations among patients with *TP53* and *ATM* comutation, 251.5 (162.0-412.3) mutations among patients with *TP53* mutation alone (*P* = .002), 205.0 (129.3-341.0) mutations among patients with *ATM* mutation alone (*P* = .003), and 122.0 (54.0-245.3) mutations among patients with no mutation (*P* < .001). Among patients in the MSKCC cohort^19^ who underwent the 341 panel NGS, median (IQR) TMB was 21.0 (10.0-26.5) mutations among patients with *TP53* and *ATM* comutation, 7.0 (4.0-12.0) mutations among patients with *TP53* mutation alone (*P* < .001), 9.0 (7.8-13.8) mutations among patients with *ATM* mutation alone (*P* = .04), and 4.0 (2.0-7.0) mutations among patients with no mutations (*P* < .001). Among patients in the MSKCC cohort^19^ who underwent the 410 panel NGS, median (IQR) TMB was 14.0 (9.0-21.0) mutations among patients with *TP53* and *ATM* comutation, 7.0 (4.0-12.0) mutations among patients with *TP53* mutation alone (*P* < .001), 8.0 (5.0-11.0) mutations among patients with *ATM* mutation alone (*P* < .001), and 4.0 (2.0-7.0) mutations among patients with no mutation (*P* < .001). In the Geneplus cohort, the median (IQR) TMB was 13.5 (6.3-23.8) mutations among patients with *TP53* and *ATM* comutation, 6.0 (4.0-10.) mutations among patients with *TP53* mutation alone (*P* < .001), 5.0 (4.0-10.3) mutations among patients with *ATM* mutation alone (*P* < .001), and 3.0 (2.0-6.0) mutations among patients with no mutation (*P* < .001). In the POPLAR^4^ and OAK^20^ cohort, the median (IQR) TMB was 19.0 (12.8-31.5) mutations among patients with *TP53* and *ATM* comutation, 11.0 (6.0-20.0) mutations among patients with *TP53* mutation alone (*P* < .001), 6.5 (3.0-14.8) mutations among patients with *ATM* mutation alone (*P* < .001), and 5.0 (3.0-9.0) mutations among patients with no mutation (*P* < .001) (Figure 2). We found a similar association of the degree of the TMB with *TP53* and *ATM* comutation and *MMR* genes, *POLE/D1,* and *BRCA1/2* mutation (eFigure 4A and B in the Supplement). In addition, driver genes, such as the *EGFR* mutation, were associated with a decreased TMB and impaired response to ICIs in patients with NSCLC.^25,26^ However, analysis of the TMB among patients with the *EGFR* mutation, *EGFR* wild type, or *TP53* and *ATM* comutation with or without *EGFR* mutation found that when an *EGFR* mutation occurred with the *TP53* and *ATM* comutation, patients still exhibited a high TMB level (eFigure 4C in the Supplement).

### Association of *TP53* and *ATM* Comutation With Response to ICIs

**Figure 3.**
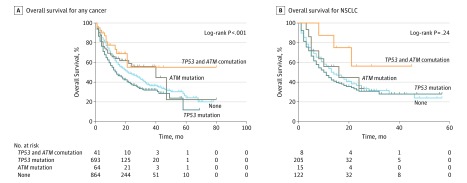
Association of *TP53* and *ATM* Mutation Type With Prognosis in Patients Treated With Immune Checkpoint Inhibitors in the Memorial Sloan Kettering Cancer Center Cohort^23^ *ATM* indicates ataxia-telangiectasia mutated gene; NSCLC, non–small cell lung cancer; *TP53*, tumor protein p53 gene; and crosses, patients who were censored.

Non–small cell lung cancer tumors among patients with the *TP53* and *ATM* comutation had a significantly increased TMB, so we used publicly available trial data to investigate whether these patients could benefit from ICIs. In the MSKCC cohort,^23^ there were 1662 patients with any cancer and 350 patients with NSCLCs who had undergone NGS and had received at least 1 dose of ICI therapy (eTable 2 in the Supplement). A total of 41 patients with any cancer and 8 patients with NSCLC specifically were found to have the *TP53* and *ATM* comutation. We found that a *TP53* and *ATM* comutation was associated with better OS than a *TP53* mutation alone, an *ATM* mutation alone, and no mutation among patients with any cancer (NSCLC median OS: *TP53* and ATM comutation, not reached; *TP53* mutation alone, 11.0 months; *ATM* mutation alone, 16.0 months; no mutation 14.0 months, *P* = .24; any cancer median OS: *TP53* and *ATM* comutation; *TP53* mutation alone, 14.0 months; *ATM* mutation alone, 40.0 months; no mutation, 22.0 months; *P* < .001) (Figure 3; eFigure 5, eTable 4, and eTable 5 in the Supplement).

**Figure 4.**
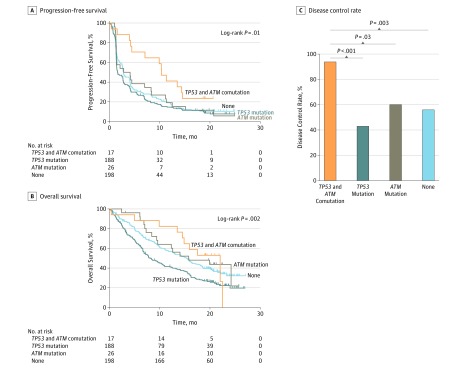
Association of *TP53* and *ATM* Mutation Type With Prognosis and Response in the POPLAR^4^ and OAK^20^ Cohort *ATM* indicates ataxia-telangiectasia mutated gene; NSCLC, non–small cell lung cancer; *TP53*, tumor protein p53 gene; and crosses, patients who were censored.

In the POPLAR^4^ and OAK^20^ cohort, a total of 429 patients received atezolizumab, and 17 patients were identified with a *TP53* and *ATM* comutation (eTable 3 in the Supplement). Disease control rate, progression-free survival, and OS were all greater in patients with the *TP53* and *ATM* comutation compared with the other 3 groups (progression-free survival: *TP53* and *ATM* comutation*,* 10.4 months; *TP53* mutation alone, 1.6 months; *ATM* mutation alone, 3.5 months; no mutation, 2.8 months, *P* = .01; median OS: *TP53* and *ATM*, 22.1 months; *TP53* mutation alone, 8.3 months; *ATM* mutation alone, 15.8 months; no mutation, 15.3 months; *P* = .002) (Figure 4A and B; eFigure 6 and eTable 6 in the Supplement). Increased progression-free survival remained statistically significant with adjustment for sex, age, Eastern Cooperative Oncology Group performance status, histologic examination results, TMB, MMR genes, *POLE/D1*, and *BRCA1/2* (hazard ratio, 0.48 [95% CI, 0.28-0.84]; *P* = .001) (eTable 7 in the Supplement).

### Gene Signatures and Pathways Associated With *TP53* and *ATM* Comutation

To further explore the distinct phenotypic and immunologic states associated with the *TP53* and *ATM* comutation, we expanded the analysis to TCGA^15^ RNA-sequence data sets using 969 samples that had paired whole-exon sequencing information. Checkpoint ligand expression of PD-L1 was significantly higher among the comutated group (FPKM, 87.1) compared with the no mutation group (FPKM, 54.7) (eFigure 7 in the Supplement). Gene set enrichment analysis based on hallmark gene sets further identified several signaling pathways that were significantly altered (false discovery rate ≤ 0.10), including higher activation of *E2F* targets, *MYC* targets, *G2M* checkpoint, *MTORC1* signaling, DNA repair, unfolded protein response, spermatogenesis, *KRAS* signaling, mitotic spindle, and hypoxia pathways in people with comutated *TP53* and *ATM* (eFigure 8 in the Supplement). Interestingly, we found that the gene set associated with angiogenesis was significantly downregulated among people with the *TP53* and *ATM* comutation compared with people with only *TP53* or *ATM* mutation and people with no mutation. However, by comparing the immune landscape among the groups, we did not observe a significant difference within any immune cell subpopulation (eFigure 7 in the Supplement).

## Discussion

In this study, we found that comutation of the DDR-related genes *TP53* and *ATM*, regardless of the *EGFR* mutation and status of other DDR-related genes, was associated with a higher TMB and an improved response to ICIs in patients with NSCLC. The p53 protein promotes either the elimination or repair of damaged cells after DNA damage and stimulates DNA repair by activating target genes that encode components of the DNA repair machinery.^27,28^ A *TP53* mutation can correlate with patterns of single-nucleotide variants and specific comutated genes.^29^ An *ATM* deficiency is likely a selected genomic aberration in multiple malignant tumors because of its protection from p53-driven apoptosis.^30,31^ Beyond mediating apoptosis, *ATM* also plays a role with *TP53* in DNA double-strand break repair^32^ and is required for efficient repair of double-strand breaks induced in heterochromatin^33^ or with blocked DNA ends.^34^ Nonhomologous end joining and homologous recombination are the 2 major pathways for the repair of double-strand breaks.^35,36^ The *TP53* and *ATM* comutation in cancer cells may lead to an homologous recombination deficiency that results in a greater dependency on nonhomologous end joining pathways. Moreover, nonhomologous end joining modifies the broken DNA ends and ligates them together with no regard for homology, generating deletions or insertions.^35,36,37^ Theoretically, *TP53* and *ATM* comutation may cause cancer cell resistance to apoptosis and thus accumulate mutations over time.

In this study, we found that some cell cycle–related pathways, such as *E2F* targets and *MYC* targets, invasive-related hypoxia, and the angiogenesis pathway were overactivated. This indicated an aggressive property and poor survival of *TP53* and *ATM* comutated tumors.^38,39,40^ We found that *TP53* and *ATM* comutation was associated with a significantly increased TMB in large independent cohorts with no difference in *POLE*, *POLD1*, or MMR genes or *BRCA1/2* mutation; PD-L1 expression was significantly upregulated in the comutation group. Furthermore, in the POPLAR^4^ and OAK^20^ cohort and the MSKCC all-cancer cohort,^19^ we found a clinical benefit and survival improvement associated with *TP53* and *ATM* comutation.

Recent studies have shown that *EGFR* mutations are associated with a low TMB, an uninflamed tumor microenvironment, and weak immunogenicity, which are associated with an inferior response to programmed cell death protein 1 and PD-L1 blockade in NSCLC.^25,41,42^ We found that patients who had *EGFR* mutations accompanied by *TP53* and *ATM* comutation still had a high TMB, but whether this small subgroup of patients would benefit from ICIs needs further confirmation in randomized clinical trials. A 2017 study by Dong et al^25^ found that GTPase (*KRAS*) (OMIM 190070) mutation, a *TP53* and *KRAS* proto-oncogene, may boost PD-L1 expression, T-cell infiltration, and augment tumor immunogenicity, resulting in a response to programmed cell death protein 1 and PD-L1 inhibitor. This indicates there may be clinical relevance for NSCLC subtyping by driver mutation genes. However, our data suggest that members of the DDR pathway, such as *TP53* and *ATM*, potentially result in genomic instability and further lead to a high TMB. This phenomenon is independent of driver mutation status and indicated there may be a subpopulation of patients who have a better chance of benefiting from ICI treatment.

This finding may have important implications for clinical practice, and we recommend *TP53* and *ATM* screening for patients with NSCLC. Even in patients with *EGFR* and other driver genes mutations, *TP53* and *ATM* comutation may be associated with an additional clinical benefit for ICI therapy.

### Limitations

Our study has limitations. Despite the *TP53* and *ATM* comutation being associated with increasing TMBs in 5 large independent NSCLC cohorts, the small number of *TP53* and *ATM* mutation tumors and few patients who received ICIs in the MSKCC^23^ and POPLAR^4^ and OAK^20^ cohorts with a recorded survival advantage were not well reflected in the Kaplan-Meier survival analysis. This indicates that our results should be interpreted with caution, and further additional prospective clinical trials of checkpoint blockade in patients with *TP53* and *ATM* comutation and NSCLC are warranted. Additionally, *TP53* and *ATM* comutation also occurred in many other cancer types, suggesting that it may be a generalized cancer phenotype (eFigure 2 in the Supplement). The mechanisms underlying the association of *TP53* and *ATM* comutation with a better prognosis for ICI treatment and a higher TMB in other cancer types are still unclear. The full implications of *TP53* and *ATM* comutation remain elusive and require further study.

## Conclusions

Our findings suggest that the *TP53* and *ATM* comutation occurs in a subgroup of patients with NSCLC and is associated with an increased TMB and response to ICIs. Comutation of *TP53* and *ATM* may have implications as a potential biomarker for guiding ICI immunotherapy.

## Notes

Supplement. **eMethods 1.** Sample Processing and DNA Extraction **eMethods 2.** Library Preparation, Target Capture and Next-Generation Sequencing **eMethods 3.** Next-Generation Sequencing Analysis **eFigure 1.** Data Sources **eFigure 2.** Incidence of *TP53* and *ATM* Comutation in the Cancer Genome Atlas and Geneplus Cohorts **eFigure 3.** Assessment of Mutation Sites in *TP53* and *ATM* Between Comutated and Solely Mutated Samples in Patients With Non–Small Cell Lung Cancer From the Cancer Genome Atlas and Geneplus Cohorts **eFigure 4.** Tumor Mutation Burden of Samples Among Patients With Non–Small Cell Lung Cancer in the Cancer Genome Atlas, Memorial Sloan Kettering Cancer Center, Geneplus, and POPLAR and OAK Cohorts **eFigure 5.** Association of *TP53* and *ATM* Mutation Type With Overall Survival in Patients Treated With Immune Checkpoint Inhibitors in the Memorial Sloan Kettering Cancer Center Cohort **eFigure 6.** Association of *TP53* and *ATM* Mutation Type With Survival Among Patients in the POPLAR and OAK Cohort **eFigure 7.** Gene Signatures Associated With *TP53* and *ATM* Comutation in Patients With Non–Small Cell Lung Cancer in the Cancer Genome Atlas Cohort **eFigure 8.** Gene Set Enrichment Analysis Identified Signaling Pathways Associated With *TP53* and *ATM* Comutation in Patients With Non–Small Cell Lung Cancer in the Cancer Genome Atlas Cohort **eTable 1.** Detailed List of Genes in the Geneplus 59 Panel and 1021 Panel **eTable 2.** Characteristics of Patients Treated With Immune Checkpoint Inhibitors in the Memorial Sloan Kettering Cancer Center Cohort **eTable 3.** Characteristics of Patients Treated With Immune Checkpoint Inhibitors in the POPLAR and OAK Cohort **eTable 4.** Univariate Analysis of Factors Associated With Survival Among Patients With Non–Small Cell Lung Cancer Treated With Immune Checkpoint Inhibitors in the Memorial Sloan Kettering Cancer Center Cohort **eTable 5.** Univariate Analysis of Factors Associated With Survival Among Patients With Any Cancer Treated With Immune Checkpoint Inhibitors in the Memorial Sloan Kettering Cancer Center Cohort **eTable 6.** Univariate Analysis of Factors Associated With Survival Among Patients Treated With Immune Checkpoint Inhibitors in the POPLAR and OAK Cohort **eTable 7.** Multivariable Analysis of Factors Associated With Survival of Patients Treated With Immune Checkpoint Inhibitors in the POPLAR and OAK Cohort **eReferences**
