# Supplementary material for: Association of Tumor Protein p53 and Ataxia-Telangiectasia Mutated Comutation With Response to Immune Checkpoint Inhibitors and Mortality in Patients With Non–Small Cell Lung Cancer
Source: JAMA Netw Open. 2019 Sep 20;2(9):e1911895. doi: 10.1001/jamanetworkopen.2019.11895 (PMC6755545; doi:10.1001/jamanetworkopen.2019.11895)
Supplement: Supplement. — eMethods 1. Sample Processing and DNA Extraction eMethods 2. Library Preparation, Target Capture and Next-Generation Sequencing eMethods 3. Next-Generation Sequencing Analysis eFigure 1. Data Sources eFigure 2. Incidence of TP53 and ATM Comutation in the Cancer Genome Atlas and Geneplus Cohorts eFigure 3. Assessment of Mutation Sites in TP53 and ATM Between Comutated and Solely Mutated Samples in Patients With Non–Small Cell Lung Cancer From the Cancer Genome Atlas and Geneplus Cohorts eFigure 4. Tumor Mutation Burden of Samples Among Patients With Non–Small Cell Lung Cancer in the Cancer Genome Atlas, Memorial Sloan Kettering Cancer Center, Geneplus, and POPLAR and OAK Cohorts eFigure 5. Association of TP53 and ATM Mutation Type With Overall Survival in Patients Treated With Immune Checkpoint Inhibitors in the Memorial Sloan Kettering Cancer Center Cohort eFigure 6. Association of TP53 and ATM Mutation Type With Survival Among Patients in the POPLAR and OAK Cohort eFigure 7. Gene Signatures Associated With TP53 and ATM Comutation in Patients With Non–Small Cell Lung Cancer in the Cancer Genome Atlas Cohort eFigure 8. Gene Set Enrichment Analysis Identified Signaling Pathways Associated With TP53 and ATM Comutation in Patients With Non–Small Cell Lung Cancer in the Cancer Genome Atlas Cohort eTable 1. Detailed List of Genes in the Geneplus 59 Panel and 1021 Panel eTable 2. Characteristics of Patients Treated With Immune Checkpoint Inhibitors in the Memorial Sloan Kettering Cancer Center Cohort eTable 3. Characteristics of Patients Treated With Immune Checkpoint Inhibitors in the POPLAR and OAK Cohort eTable 4. Univariate Analysis of Factors Associated With Survival Among Patients With Non–Small Cell Lung Cancer Treated With Immune Checkpoint Inhibitors in the Memorial Sloan Kettering Cancer Center Cohort eTable 5. Univariate Analysis of Factors Associated With Survival Among Patients With Any Cancer Treated With Immune Checkpoint Inhibitors in the Memorial Sloan Ket [file jamanetwopen-2-e1911895-s001.pdf]

Supplementary Online Content

Chen Y, Chen G, Li J, et al. Association of tumor protein p53 and ataxia-telangiectasia mutated comutation with response to immune checkpoint inhibitors and mortality in patients with non–small cell lung cancer. *JAMA Netw Open*. 2019;2(9):e1911895. doi:10.1001/jamanetworkopen.2019.11895

eMethods 1. Sample Processing and DNA Extraction
eMethods 2. Library Preparation, Target Capture and Next-Generation Sequencing
eMethods 3. Next-Generation Sequencing Analysis
eFigure 1. Data Sources
eFigure 2. Incidence of *TP53* and *ATM* Comutation in the Cancer Genome Atlas and Geneplus Cohorts
eFigure 3. Assessment of Mutation Sites in *TP53* and *ATM* Between Comutated and Solely Mutated Samples in Patients With Non–Small Cell Lung Cancer From the Cancer Genome Atlas and Geneplus Cohorts
eFigure 4. Tumor Mutation Burden of Samples Among Patients With Non–Small Cell Lung Cancer in the Cancer Genome Atlas, Memorial Sloan Kettering Cancer Center, Geneplus, and POPLAR and OAK Cohorts
eFigure 5. Association of *TP53* and *ATM* Mutation Type With Overall Survival in Patients Treated With Immune Checkpoint Inhibitors in the Memorial Sloan Kettering Cancer Center Cohort
eFigure 6. Association of *TP53* and *ATM* Mutation Type With Survival Among Patients in the POPLAR and OAK Cohort
eFigure 7. Gene Signatures Associated With *TP53* and *ATM* Comutation in Patients With Non–Small Cell Lung Cancer in the Cancer Genome Atlas Cohort
eFigure 8. Gene Set Enrichment Analysis Identified Signaling Pathways Associated With *TP53* and *ATM* Comutation in Patients With Non–Small Cell Lung Cancer in the Cancer Genome Atlas Cohort
eTable 1. Detailed List of Genes in the Geneplus 59 Panel and 1021 Panel
eTable 2. Characteristics of Patients Treated With Immune Checkpoint Inhibitors in the Memorial Sloan Kettering Cancer Center Cohort
eTable 3. Characteristics of Patients Treated With Immune Checkpoint Inhibitors in the POPLAR and OAK Cohort
eTable 4. Univariate Analysis of Factors Associated With Survival Among Patients With Non–Small Cell Lung Cancer Treated With Immune Checkpoint Inhibitors in the Memorial Sloan Kettering Cancer Center Cohort
eTable 5. Univariate Analysis of Factors Associated With Survival Among Patients With Any Cancer Treated With Immune Checkpoint Inhibitors in the Memorial Sloan Kettering Cancer Center Cohort
eTable 6. Univariate Analysis of Factors Associated With Survival Among Patients Treated With Immune Checkpoint Inhibitors in the POPLAR and OAK Cohort
eTable 7. Multivariable Analysis of Factors Associated With Survival of Patients Treated With Immune Checkpoint Inhibitors in the POPLAR and OAK Cohort

© 2019 Chen Y et al. *JAMA Network Open.*

eReferences.

This supplementary material has been provided by the authors to give readers additional information about their work.

© 2019 Chen Y et al. *JAMA Network Open.*

eMethods 1. Sample Processing and DNA Extraction

The genomic DNA (gDNA) of peripheral blood lymphocytes and frozen tissue samples was extracted by using the DNeasy Blood & Tissue Kit (Qiagen, Hilden, Germany). FFPE (formalin-fixed, paraffin-embedded) DNA was isolated by using a commercially available kit (Maxwell® 16 FFPE Plus LEV DNA Purification, Qiagen, Hilden, Germany Kit. catalog: AS1135). Circulating free DNA (cfDNA) from liquid biopsies was extracted by using QIAamp Circulating Nucleic Acid Kit (Qiagen, Hilden, Germany). The DNA concentration was measured using a Qubit fluorometer and the Qubit dsDNA HS (High Sensitivity) Assay Kit (Invitrogen, Carlsbad, CA, USA).

© 2019 Chen Y et al. *JAMA Network Open.*

eMethods 2. Library Preparation, Target Capture and Next-Generation Sequencing

Sequencing was carried out using Illumina 2 × 75-bp paired-end reads on an Illumina HiSeq 3000 instrument according to the manufacturer's recommendations using the KAPA DNA Library Preparation Kit (Kapa Biosystems, Wilmington, MA, USA). Barcoded libraries were hybridized to a customized panel of 1021 genes containing whole exons and selected introns of 288 genes and selected regions of 733 genes (eTable 1 in Supplement). The libraries were sequenced to a uniform median depth (> 500 × for gDNA, >1000 × for cfDNA) and assessed for somatic variants including single nucleotide variants (SNVs), small insertions and deletions (InDels), copy number alterations (CNA), and gene fusions/rearrangements.

© 2019 Chen Y et al. *JAMA Network Open.*

eMethods 3. Next-Generation Sequencing Analysis

MuTect2 (version 1.1.4)[1] and NChot[2] were employed to call somatic SNVs, and GATK[3] was used to call small insertions and deletions (indels). CNA was identified with Contra (v2.0.8)[4].

© 2019 Chen Y et al. *JAMA Network Open.*

eFigure 1. Data Sources

Geneplus

Chinese cohort
- NGS = 17814
- RNA-Seq = 0
- Survival Data = 0

TCGA

TCGA-NSCLC cohort
- WES = 1031
- RNA-Seq = 969
- Survival Data = 0

MSKCC

MSKCC-NSCLC cohort
- NGS = 1527
- RNA-Seq = 0
- Survival Data = 0

MSKCC-IO cohort
- NGS = 1662
- RNA-Seq = 0
- Survival Data = 1662

POPLAR/OAK

POPLAR/OAK cohort
- NGS = 853
- RNA-Seq = 0
- Survival Data = 853

© 2019 Chen Y et al. *JAMA Network Open.*

eFigure 2. Incidence of *TP53* and *ATM* Comutation in the Cancer Genome Atlas and Geneplus Cohorts

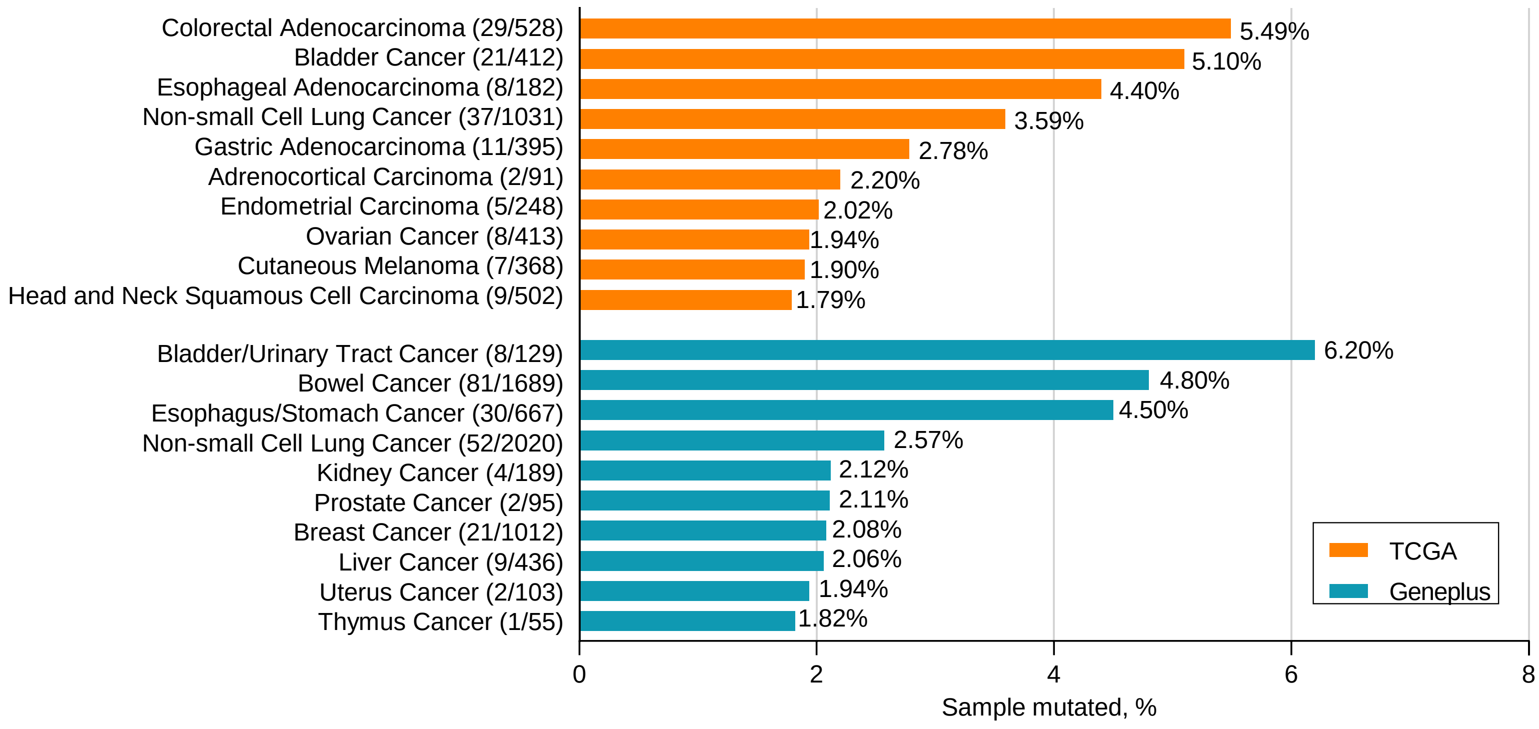

© 2019 Chen Y et al. *JAMA Network Open.*

eFigure 3. Assessment of Mutation Sites in *TP53* and *ATM* Between Comutated and Solely Mutated Samples in Patients With Non–Small Cell Lung Cancer From the Cancer Genome Atlas and Geneplus Cohorts

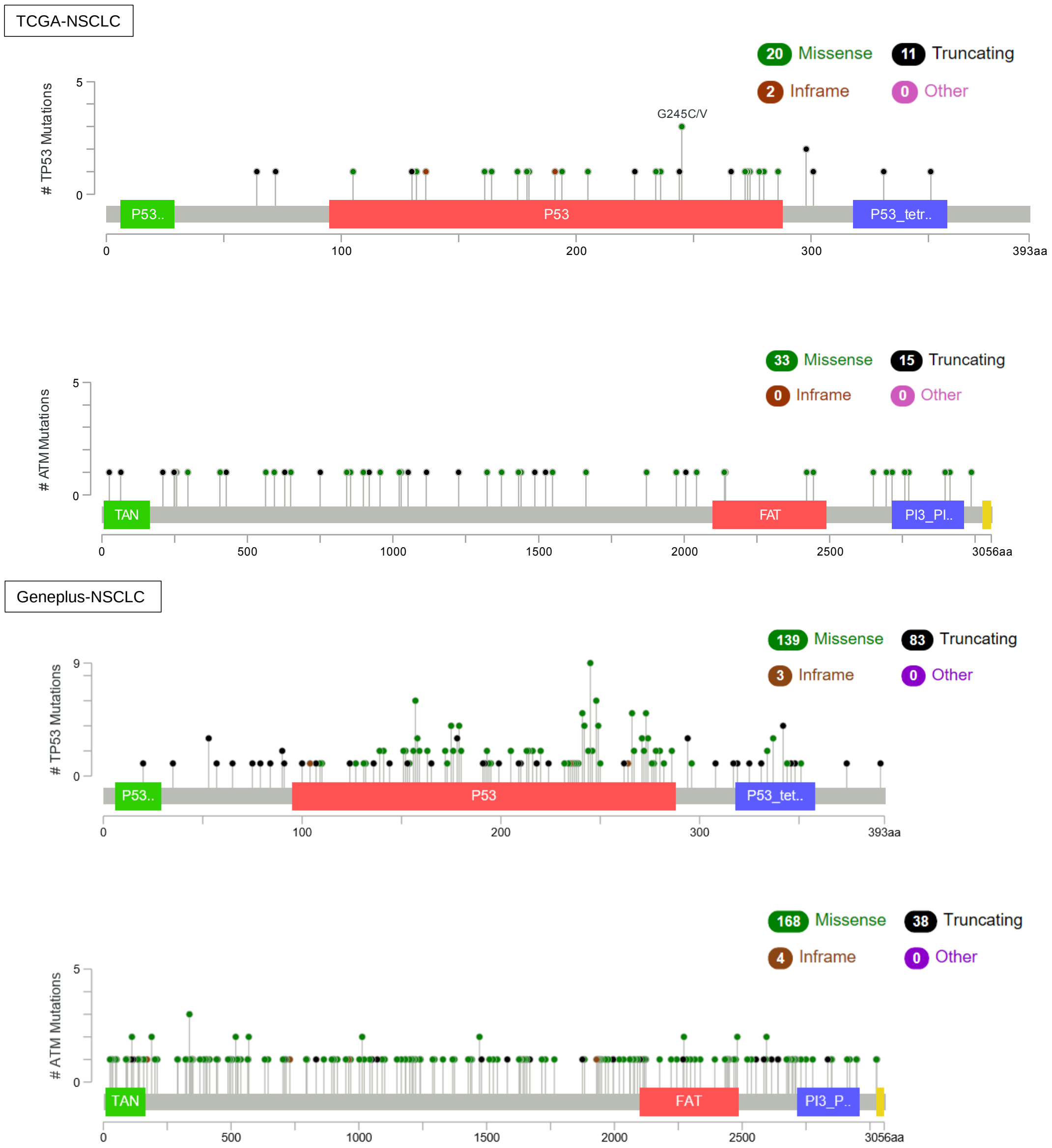

© 2019 Chen Y et al. *JAMA Network Open.*

eFigure 4. Tumor Mutation Burden of Samples Among Patients With Non–Small Cell Lung Cancer in the Cancer Genome Atlas, Memorial Sloan Kettering Cancer Center, Geneplus, and POPLAR and OAK Cohorts

(A) Comparison of tumor mutation burden of samples by *TP53/ATM* co-mutation, *BRCA1/2* mutation, MMR genes mutation, *POLE/D1* mutation in NSCLC patients from TCGA, MSKCC_341 genes, MSKCC_410 genes, Geneplus and POPLAR/OAK cohorts. (B) Comparison of tumor mutation burden of samples by *TP53/ATM* co-mutation with or without *BRCA1/2* mutation, MMR genes mutation, *POLE/D1* mutation in NSCLC patients from TCGA, MSKCC_341 genes, MSKCC_410 genes, Geneplus and POPLAR/OAK cohorts. *With other mutation: *TP53/ATM* co-mutation coexists with any type of mutation in *BRCA1/2*, MMR genes, *POLE/D1*. **Without other mutation: *TP53/ATM* co-mutation without any type of mutation in *BRCA1/2*, MMR genes, *POLE/D1*. (C) Comparison of tumor mutation burden of samples among *EGFR* mutation, *EGFR* wildtype, *TP53/ATM* co-mutation combined with or without *EGFR* mutation in NSCLC patients from MSKCC_341 genes, MSKCC_410 genes, Geneplus and POPLAR/OAK cohorts.

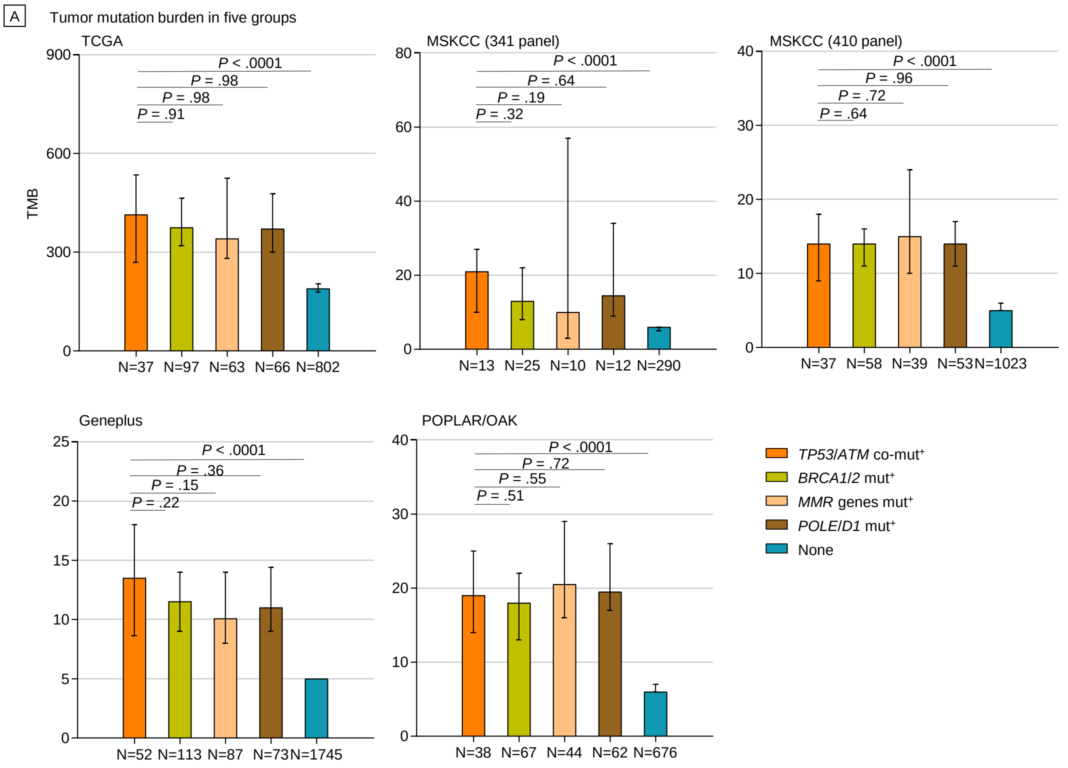

© 2019 Chen Y et al. *JAMA Network Open.*

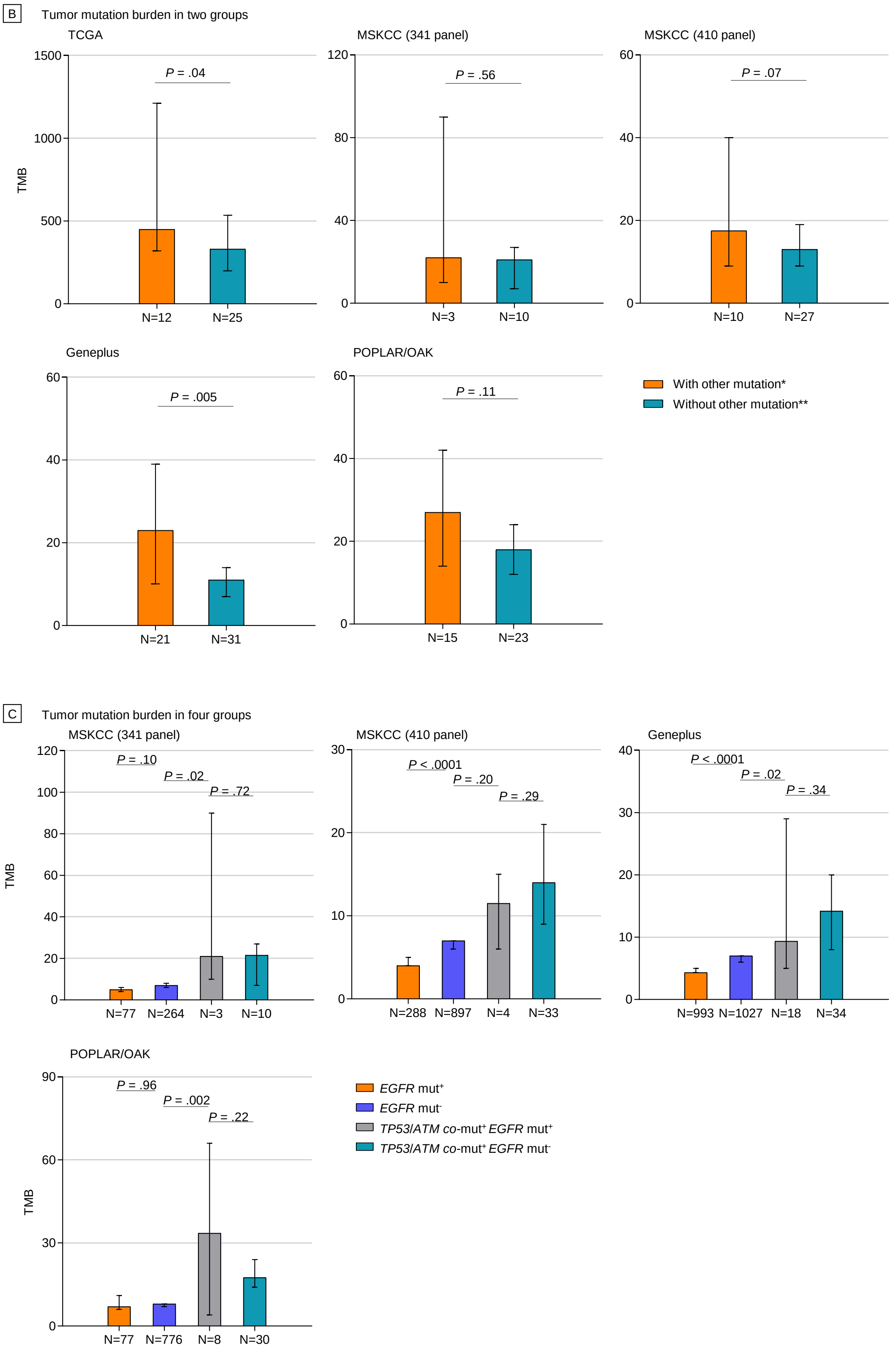

© 2019 Chen Y et al. *JAMA Network Open.*

eFigure 5. Association of *TP53* and *ATM* Mutation Type With Overall Survival in Patients Treated With Immune Checkpoint Inhibitors in the Memorial Sloan Kettering Cancer Center Cohort

Overall survival by *TP53/ATM* mutation type in MSKCC-IO pan cancer cohort (A), MSKCC-IO NSCLC cohort (B).

A

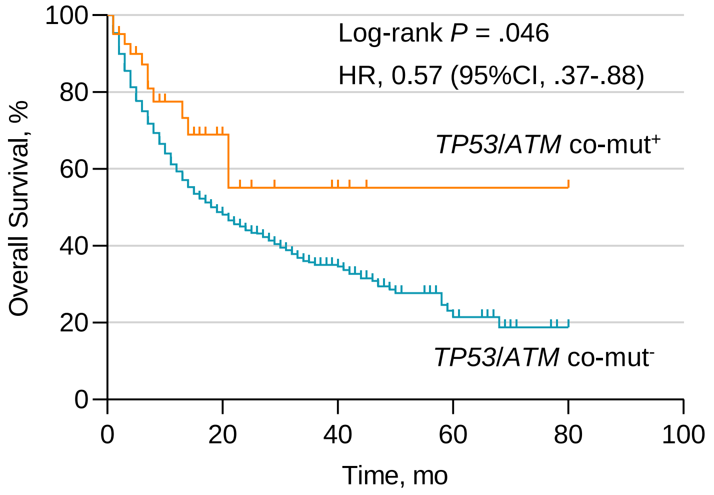

No. at risk						
TP53/ATM co-mut$^+$	41	10	5	1	0	0
TP53/ATM co-mut$^-$	1590	390	74	12	0	0

B

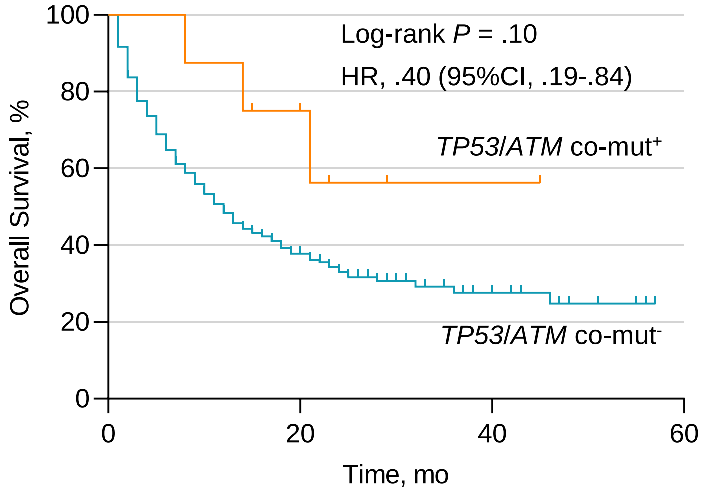

No. at risk				
TP53/ATM co-mut$^+$	8	4	1	0
TP53/ATM co-mut$^-$	342	68	13	0

© 2019 Chen Y et al. *JAMA Network Open.*

eFigure 6 Association of *TP53* and *ATM* Mutation Type With Survival Among Patients in the POPLAR and OAK Cohort

Progression-free survival (PFS) (A) and overall survival (OS) (B) by *TP53/ATM* mutation type in patients received Atezolizumab from POPLAR/OAK cohort. PFS (C) and OS (D) by *TP53/ATM* mutation type in patients received chemotherapy from POPLAR/OAK cohort.

A

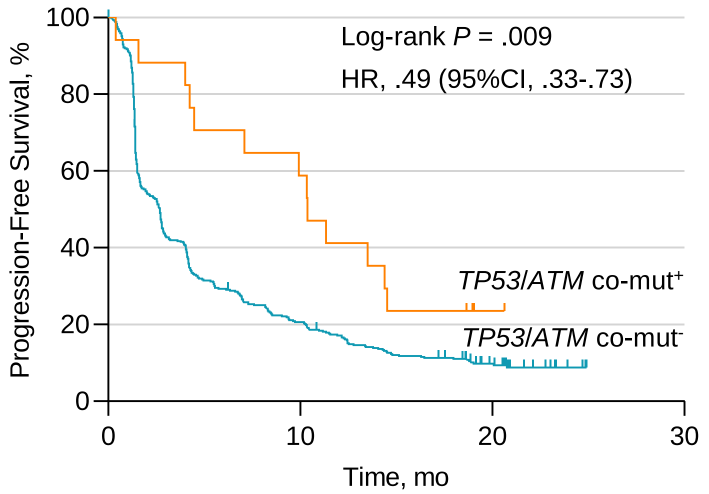

No. at risk

TP53/ATM co-mut$^+$	17	10	1	0
TP53/ATM co-mut$^-$	412	83	24	0

B

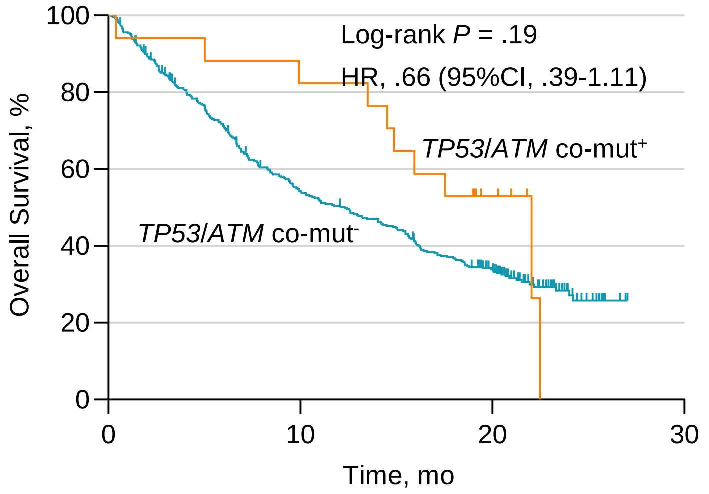

No. at risk

TP53/ATM co-mut$^+$	17	14	5	0
TP53/ATM co-mut$^-$	412	211	109	0

© 2019 Chen Y et al. *JAMA Network Open.*

C

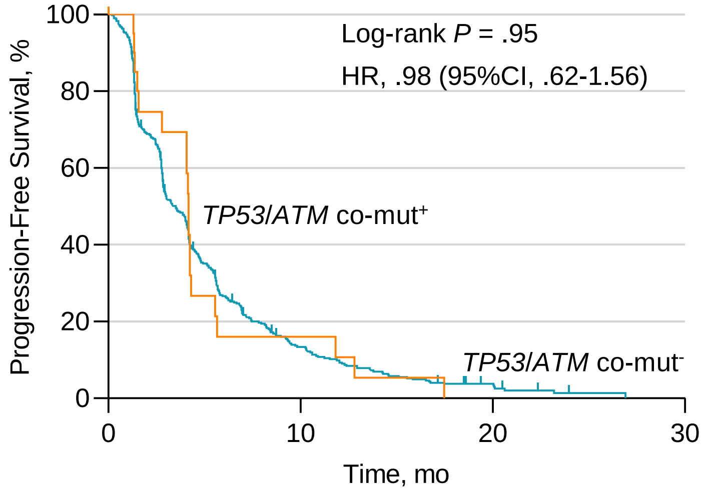

No. at risk

TP53/ATM co-mut+	21	3	0	0
TP53/ATM co-mut-	403	46	9	0

D

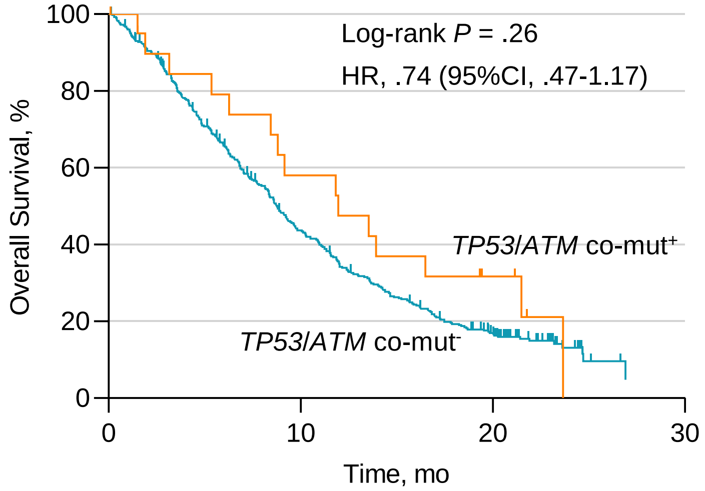

No. at risk

TP53/ATM co-mut+	21	11	4	0
TP53/ATM co-mut-	403	162	52	0

© 2019 Chen Y et al. *JAMA Network Open.*

eFigure 7. Gene Signatures Associated With *TP53* and *ATM* Comutation in Patients With Non–Small Cell Lung Cancer in the Cancer Genome Atlas Cohort

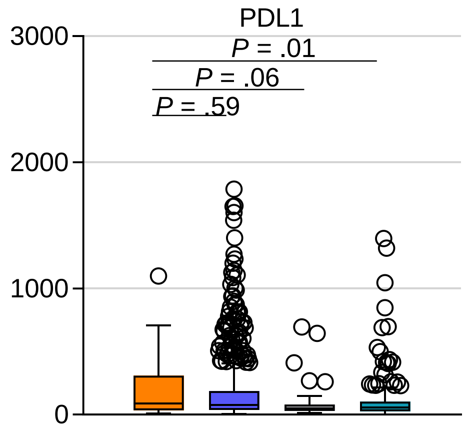

© 2019 Chen Y et al. *JAMA Network Open*.

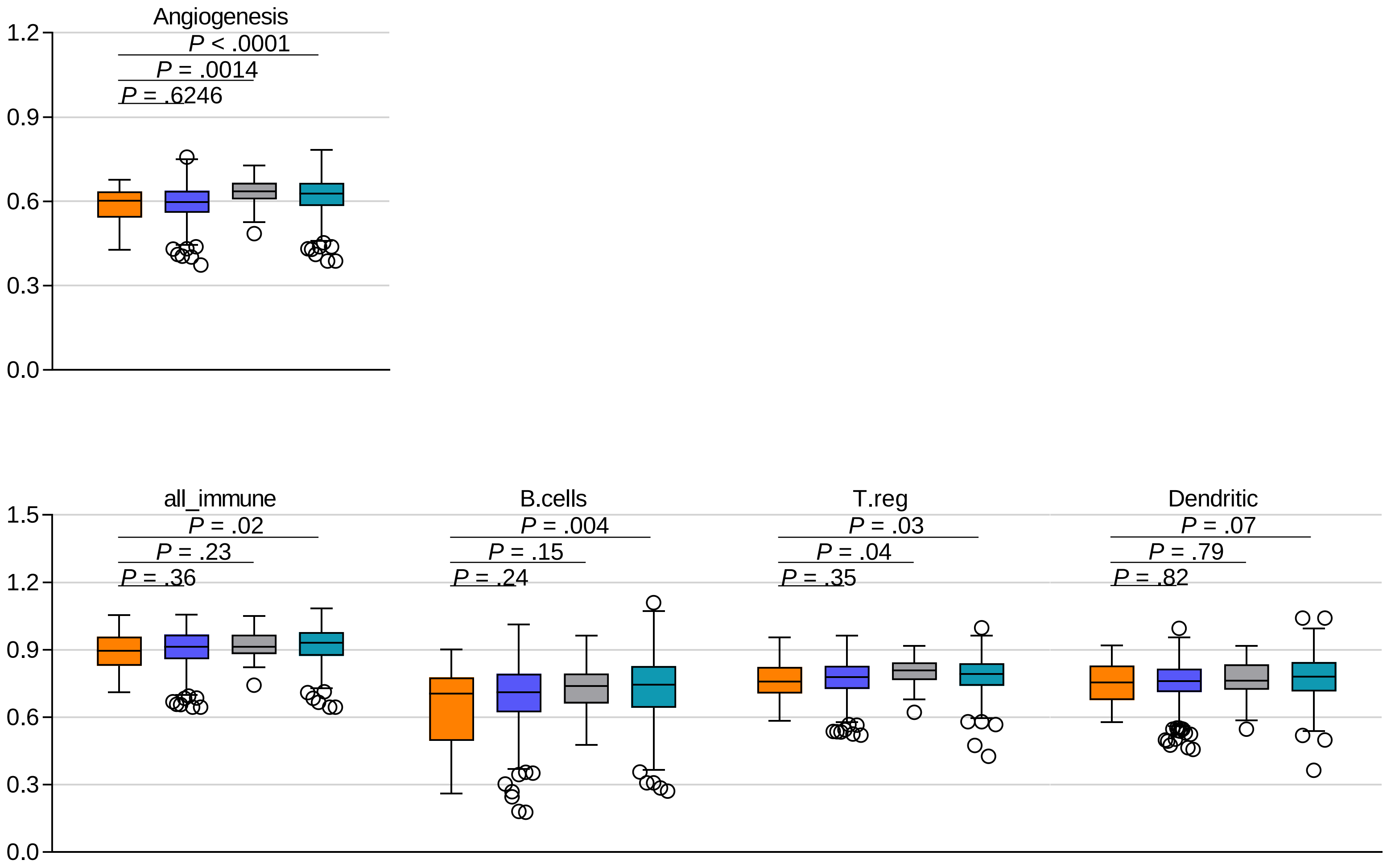

© 2019 Chen Y et al. *JAMA Network Open*.

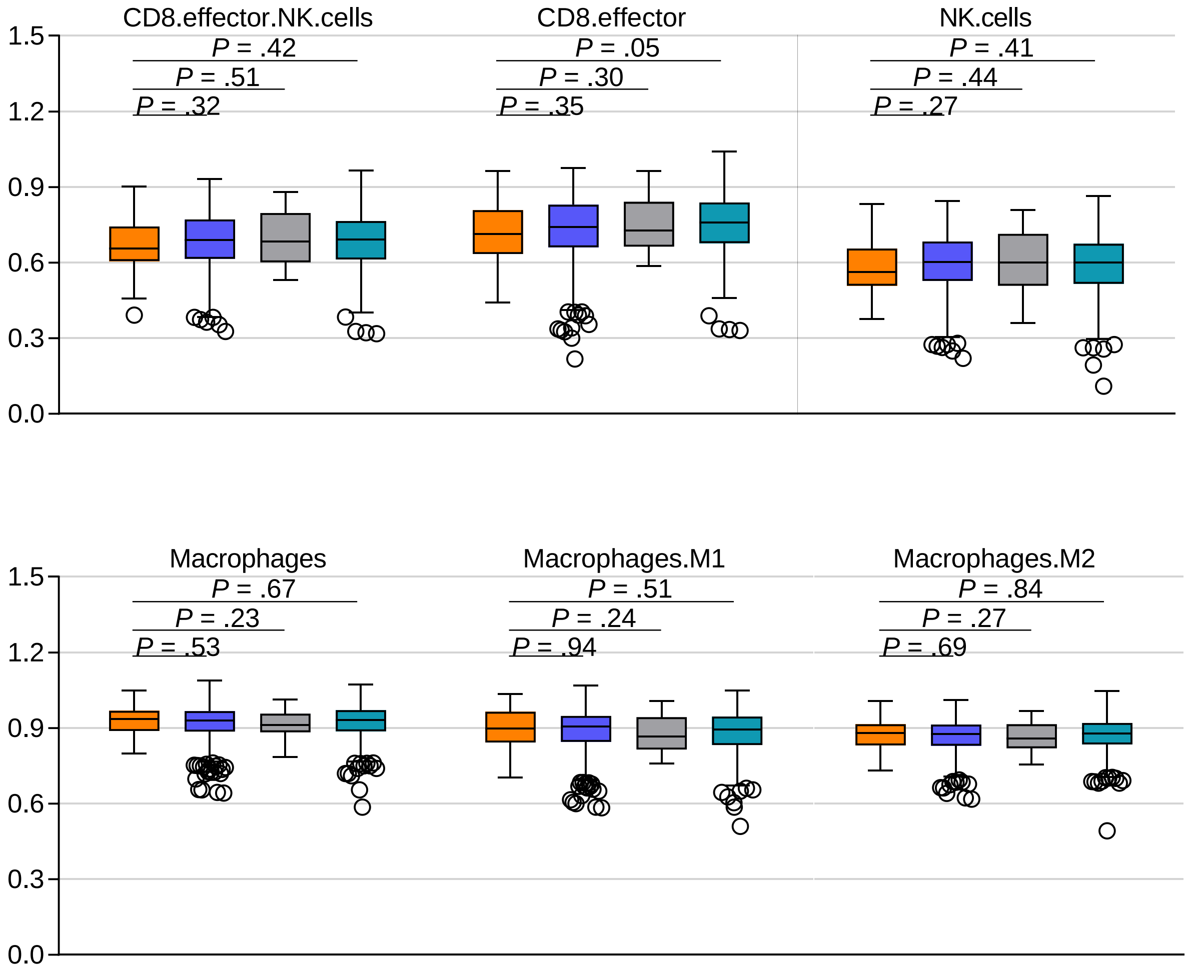

© 2019 Chen Y et al. *JAMA Network Open*.

TP53/*ATM* co-mut$^+$(*n*=34)

TP53 mut$^+$(*n*=611)

ATM mut$^+$(*n*=34)

None(*n*=290)

© 2019 Chen Y et al. *JAMA Network Open*.

eFigure 8. Gene Set Enrichment Analysis Identified Signaling Pathways Associated With *TP53* and *ATM* Comutation in Patients With Non–Small Cell Lung Cancer in the Cancer Genome Atlas Cohort

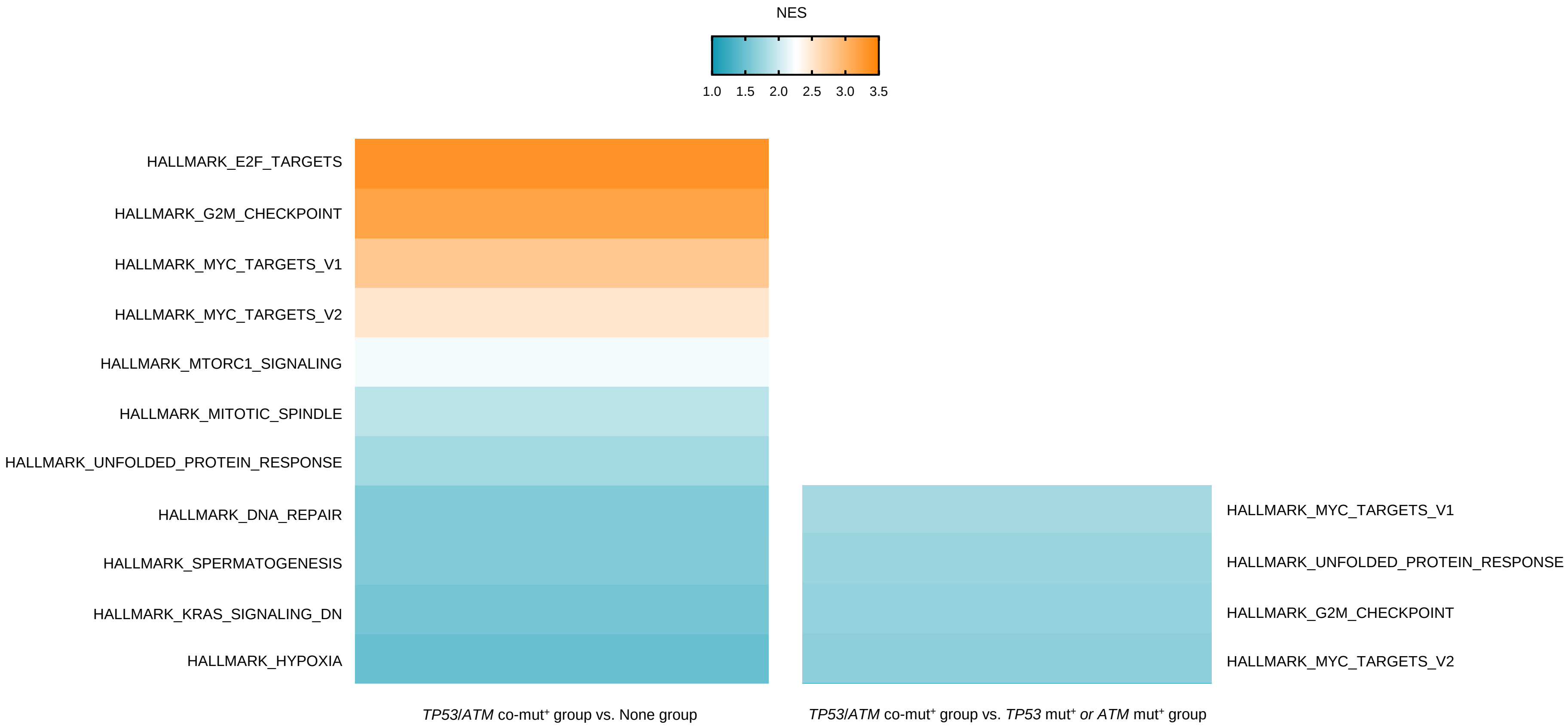

© 2019 Chen Y et al. *JAMA Network Open.*

eTable 1. Detailed List of Genes in the Geneplus 59 Panel and 1021 Panel

**Gene list of 59 panel**
AKT1 ALK APC AR ATM BCL2L11 BRAF BRCA1 BRCA2 CCND1 CDK4 CDK6 CDKN2A CTNNB1 CYP2D6 DDR2 EGFR ERBB2 ESR1 FBXW7 FGFR1 FGFR2 FGFR3 FLT3 HRAS IDH1 IDH2 JAK2 KIT KRAS MAP2K1 MAP2K2 MET MLH1 MSH2 MSH6 MTOR NF1 NRAS NTRK1 PDGFRA PIK3CA PMS2 PTCH1 PTEN PTPN11 RAF1 RB1 RET ROS1 SMARCA4 SMO SRC STK11 TP53 TSC1 TSC2 UGT1A1 VHL

**Gene list of 1021 panel**
ABL1 ABL2 AKT1 AKT2 AKT3 ALK APC AR ARAF ATM ATR AURKA AURKB AXL BAP1 BCL2 BRAF BRCA1 BRCA2 BRD2 BRD3 BRD4 BTK C11orf30 C1QA C1S CBL CCND1 CCND2 CCND3 CCNE1 CD274 CDH1 CDK13 CDK4 CDK6 CDK8 CDKN1A CDKN1B CDKN2A CDKN2B CHEK1 CHEK2 CRKL CSF1R CTNNB1 DDR1 DDR2 DNMT3A EGFR EPHA2 EPHA3 EPHA5 ERBB2 ERBB3 ERBB4 ERCC1 ERG ESR1 EZH2 FAT1 FBXW7 FCGR2A FCGR2B FCGR3A FGFR1 FGFR2 FGFR3 FGFR4 FLCN FLT1 FLT3 FLT4 FOXA1 FOXL2 GAB2 GATA3 GNA11 GNAQ GNAS HDAC1 HDAC4 HGF HRAS IDH1 IDH2 IGF1R IL7R INPP4B IRS2 JAK1 JAK2 JAK3 KDR KIT KRAS MAP2K1 MAP2K2 MAPK1 MAPK3 MCL1 MDM2 MDM4 MED12 MET MITF MLH1 MLH3 MPL MS4A1 MSH2 MSH3 MSH6 MTOR MYC MYD88 NF1 NF2 NOTCH1 NOTCH2 NOTCH3 NOTCH4 NRAS NTRK1 NTRK3 PALB2 PDGFRA PDGFRB PDK1 PIK3CA PIK3CB PIK3R1 PIK3R2 PMS1 PMS2 PRKAA1 PSMB1 PSMB5 PTCH1 PTCH2 PTEN PTPN11 RAF1 RARA RB1 RET RHEB RHOA RICTOR RNF43 ROCK1 ROS1 RPS6KB1 SMARCA4 SMARCB1 SMO SRC STAT1 STAT3 STK11 SYK TMPRSS2 TOP1 TP53 TSC1 TSC2 VEGFA VHL XPO1 XRCC1 KEAP1 NFE2L2 REG1B TPTE CSMD3 FAM135B U2AF1 THSD7A MLL3 EYA4 HCN1 AKR1B10 SLC6A5 DPP10 SCN7A SNTG1 VPS13A IL1RAPL1 CTNNA2 FAM5C CACNA1E KRTAP5-5 PDE1C RYR2 NRXN1 COL19A1 LRP1B GKN2 CD5L SPTA1 DHX9 ADAMTS20 NLRP4 CDH18 MYH2 OR5L2 OR4A15 OR6F1 OR4C6 OR2T4 PSG2 ITM2A TNN OCA2 CNTN5 POM121L12 LRRC7 CNTNAP5 SLC4A10 GFRAL SORCS3 POTEG F9 SLC26A3 UNC5D PDE4DIP MRPL1 COL25A1 TNR GALNT13 EIF3E SLC5A1 COASY TBX15 PYHIN1 PSG5 BTRC MDGA2 GUCY1A3 TIMD4 AK5 ODZ3 COL5A2 NTM LTBP1 PRSS1 CNGB3 SI TMEM132D ASTN1 SAGE1 ADAMTS12 EPB41L4B POLR3B ATP10B CSMD1 FBN2 EXOC5 ANKRD30A TRIML1 POLDIP2 KLHL1 TRIM58 GRIA3 CNOT4 NAV3 TRPC5 LRRC2 ADAMTS16 ACER2 AMOT OBP2A INHBA PTPRD FAM21A RUNX1 FAM157B SLC8A1 CBFB C9orf43 TBP NBPF10 NRXN2 TAF1B PTCD3 ACTL6B SH3PXD2A PRKAG3 UCK2 DLST MEFV FDCSP COPA LMBR1L CD99 RBMX ZDHHC11 MGAM COL16A1 PPA1 APLP2 ELL3 LILRB3 KIR2DL3 ENTPD6 BAX ANKRD36B LRP2 SF3B1 CEACAM20 C19orf38 TBX3 UMOD LPHN3 FRG1 IFT172 ZBTB8OS TNNT1 TNFAIP6 USP12 SGIP1 TEX35 WASL NWD1 MAP4K1 MAPRE3 HAAO SEC14L4 CD9 PIWIL1 CLEC16A DPP4 GMDS ST18 DKC1 FOLH1 SPAG16 CACNA1D CSPP1 PAGE1 BRWD3 CDH24 ABCA8 TMC2 EFHA2 HAUS6 WLS TNFSF4 DDB1 CTSF TMX3 MICALL1 NUDCD2 PRKDC PREX2 BCAS2 THOC1 LILRB4 PGAP1 CPA1 FAM3A RPL22 NBPF1 RYR3 PTPLAD1 FNDC4 METTL5 CAMKK1 NCOR1 COL1A1 VEZF1 KLHL14 PDRG1 VILL COL6A6 DMXL1 ADAMTS19 SYCP2L EPB41L2 TNS3 IKBKAP COL5A1 FATE1 MORN1 MAEL SLC38A4 ATP6V0A2 CASC4 TIMP3 DOCK3 KDM6A CASQ2 CDK18 FRMD4A TRUB1 SLCO1B7

© 2019 Chen Y et al. *JAMA Network Open.*

Gene list of 1021 panel
TUBGCP5 NLRC3 CTCF NOS2 POLRMT PIP5K1C AP1B1 CRTAP SIK3 ARHGEF1 MYBPC2 DZANK1 EIF3I SSBP3 TRMT112 SF1 NOS1 SETD1B TGDS ARHGEF7 LMAN1L QRICH2 EXOC4 ASH2L ATP8B2 CCT3 EXOC6 PSMC6 TRIP11 TMEM87A ZNF563 CCDC155 MAP2 SCN10A RBM6 CSN3 PDCD6 FAM114A2 LARP1 SYNE1 NACAD SLC35B4 IL11RA WDR44 ITLN2 FRMPD2 CAPRIN1 TUBGCP4 MYH8 CD300LF EXOC7 NARF CHD6 L3MBTL1 KCNQ2 RAC2 XPO5 TBL1X HDAC6 PQBP1 VSIG4 SGPL1 PDE2A GUCY2C PIP4K2C GLYR1 MYH4 SSH2 TBC1D3 ASXL2 TMEM247 BCAS1 CYTH4 MAPKAPK3 EPHB1 PTPN13 GINS4 SLCO5A1 RFX3 GABRD DNAJC11 CHI3L1 GPATCH2 FAM177B LRP4 ANKRD13D RAB6A ALDH2 PARP4 REC8 ULK3 ALDH1A3 HAP1 SDK2 CBX4 TCF4 KLK1 STAT4 PLCB4 RNF215 ZC3H7B BBS9 EIF4H PLOD3 TBX22 RPL36A GAB3 KIF1B POLR3GL NOP2 CHD4 AP1G2 CATSPER2 WDR72 UNC13D ZNF414 CACNA1A SUPT5H FLT3LG KIR3DL3 GALNT14 STK11IP DNTTIP1 CNTN4 TBC1D5 GOLGA4 ATP2C1 KIAA0922 HEATR7B2 CLINT1 MRPS18B TCP11 PKHD1 PLEC ANKRD20A4 OBP2B COL4A6 PRRX1 ETNK2 ITPKB TARBP1 MUC5B SLC17A6 NAT10 TPH2 PACS2 TBC1D21 CLCN7 PKD1L2 GPS2 CTIF HMHA1 COL5A3 ELAVL3 RPS5 FSHR ADAM33 MYLK2 SLC25A1 HPS4 EIF4ENIF1 EFCAB6 MORC1 ANK2 FNIP2 RAPGEF2 ACSL1 HIST1H3B CDK19 MED23 SGCZ TEK C9orf114 PAEP DGKK SERPINA7 IL13RA2 RERE DHDDS EPS8L3 TRIM33 IKBKE FRG2B PPFIBP2 ATXN2 HECTD4 ZC3H13 DDX24 PSTPIP1 FSD2 SULT1A4 GPR114 RPTOR SH2D3A ZAP70 IQCA1 AQP12A C20orf112 INPP5J ITIH1 MYL5 ZFR BTNL3 KIFC1 PPP1R17 HECW1 TAF6 FASTK DMD SLC38A5 WAS MTM1 CDK11A KCNAB2 WDTC1 CYP4A11 THEM5 IGSF9 AKR1C1 GAD2 ZSWIM8 LIPN ARNTL MTA2 C12orf5 PLCZ1 MYL6 DEPDC4 SPPL3 SPG20 TMTC4 TFDP1 TMED8 HOMER2 SF3B3 SUPT6H CISD3 DOT1L TYK2 CCDC159 IL27RA UNC13A ANKRD27 LGALS13 TMEM145 U2AF2 FAM49A LCT THSD7B TTN SAG PHACTR3 PPDPF PCNT ITGA9 HPS3 ATP10D IBSP UGT8 PEX6 SLC35B2 COL9A1 EEF1A1 CASP8AP2 TCP10 THBS2 CNTNAP3B PTBP3 USP48 RCC1 ATXN7L2 PI4KB CGN RFWD2 NCF2 ITGA8 DRGX FAM13C ABCC8 GYLTL1B NXF1 SIDT2 ANO2 KRT2 ACSS3 TPCN1 TESC MSI1 SLC25A30 MYCBP2 LPCAT4 SPPL2A ACSM5 KIAA0195 P4HB BSG MAST1 CYP2A13 DYSF GPAT2 NCKAP1 ARHGAP40 CDH26 ARFRP1 NUP210 COL6A5 ACPP EIF2B5 PLAC8 TLL1 NIPBL EFNA5 LRRD1 SSPO ZNF705G ZNF705B FAM49B GPR144 FRMPD4 CNKSR2 LUZP4 AGMAT ZMYM4 CCDC17 UBE2Q1 C1orf35 KIF26B AHCTF1 NELL1 ALX4 FMNL3 APAF1 POLE ALG5 UCHL3 LRRC16B SPRED1 SPINT1 DUOX1 KIAA1199 CHD3 KRT9 NT5C3L RUNDC3A ST6GALNAC1 ANKRD30B VAV1 TSKS ZNF350 ZNF614 HECW2 TGM2 ARFGAP1 ADAMTS5 SIM2 SMTN TCF20 PRKCD CRYBG3 MTTP DNAH5 TRIO SLC30A5 MAN2A1 SIM1 GIGYF1 POLR2J ENPP2 COL14A1 TYRP1 LRSAM1 MID1 MBTPS2 FUNDC1 COL4A5 F8 RPS6KA1 TTF2 ZNF687 PKLR C1orf112 HMCN1 SHISA4 MTR MYO3A SLC43A1 BLOC1S1 SLC17A8 APPL2 TMEM120B SPATA13 ATP12A RNF219 TGM5 PDILT WWP2 HYDIN DNAH9 PTGES3L-AARSD1 ABCA10 PTPRM RALBP1 SAFB2 CD97 WDR62 GIPR ARHGAP35 CNOT3 PREB KIAA1211L NMI SCN9A HSPD1 C2orf62 PPIL2 MYH9 CADM2 PDIA5 TBC1D1 SLC4A4 METTL14 LRBA SEMA6A KIAA1191 HLA-DRB5 HLA-DRB1 USP45 SYNJ2 LRRC72 ZNF804B C7orf53 TSPAN12 FLNC FAM86B1 EIF2C2 ADAMTSL1 AGTPBP1 FANCC ABCB7 NXF5 DOCK11 SLC45A1 PABPC4 EFCAB7 PRUNE CD1E

© 2019 Chen Y et al. *JAMA Network Open.*

Gene list of 1021 panel
SELP PCNXL2 DNAJC9 TMEM80 ZNF143 GLB1L3 ITFG2 OVCH1 CNTN1 ZNF385A STAT6 CPSF6 ZFC3H1 OTOGL WSCD2 GPR133 XPO4 PCK2 KTN1 SYNE2 BRF1 FAN1 FAH VPS33B OTOA FLOT2 HID1 MYOM1 MIER2 PSMC4 ZNF541 CPSF3 SF3B14 USP39 SCN3A CASP8 MLPH PTPRA APMAP HNF4A SH3BGR CECR2 ITPR1 RARB LRRFIP2 COPG1 SLC2A2 MCF2L2 KIAA0226 SEC24B DIAPH1 RREB1 KIF13A TPMT GRIK2 MYB CDK14 LUC7L2 ATG9B PSIP1 PPEF1 EIF1AX TSR2 ZNF711 STAG2 UBE4B CELA2B KDM4A ACOT11 DHCR24 FUBP1 MCOLN2 DCST1 MRPL24 KIFAP3 PTGS2 RAB3GAP2 FMN2 PHYH SAMD8 BRSK2 CARS TRIM51 STX3 MUS81 RAB1B CRTAM RPUSD4 FOXJ2 ETV6 GNPTAB WDR66 DNAH10 NCOR2 TUBA3C ESD NEK5 CARS2 MBIP PPP4R4 TP53BP1 DMXL2 LCTL SEPT12. EFCAB5 CCT6B ACE KCNH6 TMEM104 SEH1L TJP3 TIMM44 PRKACA HAUS5 ATAD2B VIT USP34 ADD2 TSGA10 C2orf47 ADAM23 HSPA12B SLC13A3 ZNF512B UBASH3A XBP1 CSNK1E RRP7A ARPC4-TTLL3 ANKRD28 OSBPL10 MAGI1 MYH15 WDR52 PLXNA1 PCYT1A LETM1 GPR125 CDS1 BMPR1B DCLK2 PLK2 AP3B1 EDN1 KIAA0319 IMPG1 MDN1 ECHDC1 SGK1 PARK2 STK31 PCLO CYP3A4 SRRT EMID2 RELN NUP205 FAM131B ABCF2 EXTL3 PHF20L1 PCSK5 TLE1 RASEF LCN10 PRKX DDX3X NLGN3 MAP2K4 MAP3K1 SETD2 KMT2B CDK12 KMT2C NUTM1 BCR KIF5B BCL2L11 TERT

© 2019 Chen Y et al. *JAMA Network Open.*

eTable 2. Characteristics of Patients Treated With Immune Checkpoint Inhibitors in the Memorial Sloan Kettering Cancer Center Cohort

**Characteristics**	***TP53/ATM* co-mut⁺ (N=41)**	***TP53/ATM* co-mut⁻ (N=1621)**	**χ2**	***P***
Sex			3.21	.073
Male	31	1003		
Female	10	618		
Age			.03	.87
≤60	18	732		
>60	23	889		
Cancer type			-	.23
NSCLC	8	342		
Melanoma	4	317		
Not NSCLC/Melanoma	29	962		
Drug class			-	.34
PD-1/PDL-1	35	1272		
CTLA-4	3	96		
Combo	3	253		
Year of ICI start			-	.89
2011-2012	1	25		
2013-2014	5	184		
2015-2017	35	1412		
Normalized mutation count of each histology			55.00	.000
Top 20%	30	372		
Bottom 80%	11	1249		
BRCA1/2			14.29	.000
Mut⁺	10	128		
Mut⁻	31	1493		
POLE/D1				
Mut⁺	9	107	14.51	.000
Mut⁻	32	1514		
MMR genes			40.63	.000
Mut⁺	13	101		
Mut⁻	28	1520		

© 2019 Chen Y et al. *JAMA Network Open.*

eTable 3. Characteristics of Patients Treated With Immune Checkpoint Inhibitors in the POPLAR and OAK Cohort

**Characteristics**	***TP53*/*ATM* co-mut$^+$ (N=17)**	***TP53*/*ATM* co-mut$^-$ (N=412)**	**$\chi 2$**	***P***
Sex			-	.13
Male	14	261		
Female	3	151		
Age			.63	.43
<65	8	234		
≥65	9	178		
Race			-	.23
White	15	289		
Asian	2	84		
Other	0	39		
ECOGGR			.12	.73
0	5	138		
1	12	274		
Histology			.33	.57
Non-squamous	11	293		
Squamous	6	119		
TMB			24.51	.000
≤16	5	330		
>16	12	82		
BRCA1/2			-	.13
Mut$^+$	3	30		
Mut$^-$	14	382		
POLE/D1				
Mut$^+$	3	30	-	.13
Mut$^-$	14	382		
MMR genes			-	.05
Mut$^+$	3	19		
Mut$^-$	14	393		

© 2019 Chen Y et al. *JAMA Network Open.*

eTable 4. Univariate Analysis of Factors Associated With Survival Among Patients With Non–Small Cell Lung Cancer Treated With Immune Checkpoint Inhibitors in the Memorial Sloan Kettering Cancer Center Cohort

**Variable**		**N**	**mOS(month)**	***P***
Sex	Male	170	10.00	.23
	Female	180	12.00	
Age	≤60	109	13.00	.29
	>60	241	11.00	
Drug class	PD-1/PDL-1	329	11.00	.01
	Combo	21	46.00	
Year of ICI start	2013-2014	34	-	.000
	2015-2017	316	10.00	
TMB of each histology	Top 20%	96	19.00	.001
	Bottom 80%	254	10.00	
BRCA1/2	Mut^+	25	17.00	.10
	Mut^-	325	11.00	
POLE/D1	Mut^+	19	-	.04
	Mut^-	331	11.00	
MMR genes	Mut^+	17	18.00	.72
	Mut^-	333	11.00	
TP53/ATM	$Co\text{-}mut^+$	8	-	.08
	$Co\text{-}mut^-$	342	11.00	

© 2019 Chen Y et al. *JAMA Network Open.*

eTable 5. Univariate Analysis of Factors Associated With Survival Among Patients With Any Cancer Treated With Immune Checkpoint Inhibitors in the Memorial Sloan Kettering Cancer Center Cohort

**Variable**		**N**	**mOS(month)**	***P***
Sex	Male	1034	19.00	.08
	Female	628	15.00	
Age	≤60	750	19.00	.95
	>60	912	18.00	
Cancer type	NSCLC	350	11.00	.43
	Melanoma	321	42.00	
	Not NSCLC/Melanoma	991	16.00	
Drug class	PD-1/PDL-1	1307	15.00	.000
	CTLA-4	99	40.00	
	Combo	256	41.00	
Year of ICI start	2011-2012	26	60.00	.000
	2013-2014	189	41.00	
	2015-2017	1447	15.00	
Normalized mutation count of each histology	Top 20%	402	34.00	.000
	Bottom 80%	1260	15.00	
BRCA1/2	Mut$^+$	138	28.00	.03
	Mut$^-$	1524	18.00	
POLE/D1	Mut$^+$	116	34.00	.01
	Mut$^-$	1546	18.00	
MMR genes	Mut$^+$	114	59.00	.004
	Mut$^-$	1548	17.00	
TP53/ATM	Co-mut$^+$	41	-	.04
	Co-mut$^-$	1621	18.00	

© 2019 Chen Y et al. *JAMA Network Open.*

eTable 6. Univariate Analysis of Factors Associated With Survival Among Patients Treated With Immune Checkpoint Inhibitors in the POPLAR and OAK Cohort

**Variable**		**N**	**mPFS(month)**	***P***	**mOS(month)**	***P***
Sex	Male	275	2.168	.0932	10.94	.08
	Female	154	2.990		15.67	
Age	<65	242	2.710	.9306	12.42	.74
	≥65	187	2.760		13.24	
Race	White	304	2.694	.5085	11.07	.01
	Asian	86	2.793		18.69	
	Other	39	2.793		11.66	
ECOGGR	0	143	3.943	.0157	17.15	<.0001
	1	286	2.530		9.626	
Histology	Not-squamous	304	2.727	.4567	15.05	.007
	Squamous	125	2.530		8.444	
TMB	≤16	335	2.694	.226	12.550	.66
	>16	94	2.858		13.503	
MMR genes	Mut^+	22	2.793	.9247	14.06	.39
	Mut^-	407	2.727		12.62	
POLE/D1	Mut^+	33	5.749	.0053	19.52	.09
	Mut^-	396	2.694		12.42	
BRCA1/2	Mut^+	33	3.023	.2709	12.32	.26
	Mut^-	396	2.727		12.78	
TP53/ATM	$Co\text{-}mut^+$	17	10.38	.0086	22.05	.19
	$Co\text{-}mut^-$	412	2.694		12.32	

© 2019 Chen Y et al. *JAMA Network Open.*

eTable 7. Multivariable Analysis of Factors Associated With Survival of Patients Treated With Immune Checkpoint Inhibitors in the POPLAR and OAK Cohort

**Variable**	**PFS (month)**			**OS (month)**		
	P	Hazard ratio	95%CI	*P*	Hazard ratio	95%CI
TP53/AMT						
Co-mut+	.01	.48	.28.84			
Sex						
Female	.03	1.26-1.02				
ECOGGR						
0	.03	1.27	1.02			
POLE/D1						
Mut-	.02	.60	.40-.91			
Race						
White (Reference)						
Asian				.004	.62	.45-.85
Other				.66	1.09	.74-1.62
ECOGGR						
1				<.0001	1.79	1.38-2.32
Histology						
Squamous				.009	1.40	1.09-1.79

© 2019 Chen Y et al. *JAMA Network Open.*

eReferences

1. Kristian C, Lawrence MS, Carter SL, et al. Sensitive detection of somatic point mutations in impure and heterogeneous cancer samples. *Nature Biotechnology.* 2013;31(3):213-219.
2. Yang X, Chu Y, Zhang R, et al. Technical Validation of a Next-Generation Sequencing Assay for Detecting Clinically Relevant Levels of Breast Cancer-Related Single-Nucleotide Variants and Copy Number Variants Using Simulated Cell-Free DNA. *The Journal of molecular diagnostics : JMD.* Jul 2017;19(4):525-536.
3. Walker MA, Pedamallu CS, Ojesina AI, et al. GATK PathSeq: a customizable computational tool for the discovery and identification of microbial sequences in libraries from eukaryotic hosts. *Bioinformatics (Oxford, England).* Dec 15 2018;34(24):4287-4289.
4. Li J, Lupat R, Amarasinghe KC, et al. CONTRA: copy number analysis for targeted resequencing. *Bioinformatics.* 2012;28(10):1307-1313.

© 2019 Chen Y et al. *JAMA Network Open.*
